# Serum gamma-glutamyltransferase activity and Parkinson’s disease risk in men and women

**DOI:** 10.1038/s41598-020-58306-x

**Published:** 2020-01-27

**Authors:** Dallah Yoo, Ryul Kim, Yu Jin Jung, Kyungdo Han, Cheol Min Shin, Jee-Young Lee

**Affiliations:** 1 Department of Neurology, Seoul National University-Seoul Metropolitan Government Boramae Medical Center, Seoul, Republic of Korea; 2 Department of Neurology, Seoul National University College of Medicine, Seoul, Republic of Korea; 3 Department of Neurology, Daejeon St. Mary’s Hospital, College of Medicine, The Catholic University of Korea, Daejeon, Republic of Korea; 4 Department of Medical Statistics, College of Medicine, The Catholic University of Korea, Seoul, Republic of Korea; 5 Department of Internal Medicine, Seoul National University Bundang Hospital, Seongnam, Gyeonggi Republic of Korea

**Keywords:** Parkinson's disease, Risk factors

## Abstract

We evaluated serum gamma-glutamyltransferase (GGT) and the risk of Parkinson’s disease (PD). Using data from the National Health Insurance Service (NHIS) database, we constructed a cohort consisting of individuals aged above 40 years who underwent a health check-up in 2009. After excluding individuals with heavy alcohol consumption, hepatobiliary and pancreatic disorders, and a previous history of PD, each quartile group of baseline serum GGT levels was monitored for the development of PD for 7 years. Adjusted hazard ratios (HRs) for PD were estimated by Cox proportional hazard models adjusting for potential confounding variables. We additionally analyzed the possible interaction between GGT and obesity or metabolic syndrome. Among the 6,098,405 individuals who were included, PD developed in 20,895 individuals during the follow-up (0.34%, 9,512 men and 11,383 women). The top quartile of serum GGT (geometric means, 90.44 IU/L in men and 41.86 IU/L in women) was associated with a lower risk in men (adjusted HR = 0.72 (95% CI: 0.67–0.76)) and a higher risk in women (adjusted HR = 1.30 (95% CI: 1.23–1.37)) using the lower GGT quartiles as a reference. Obesity and metabolic syndrome increased PD risk in both sexes, and there was only a subadditive interaction between serum GGT and obesity in women.

## Introduction

Gamma-glutamyltransferase (GGT) is mainly located in the membrane of hepatocytes and is a marker of various disease conditions affecting the liver and hepatobiliary system^1,2^. Recently, the serum level of GGT has also been reported to be associated with vascular diseases, including ischemic heart disease and stroke, as well as with metabolic syndrome and dementia^3–8^. As GGT catalyzes the transfer of glutamyl residue from glutathione to an amino acid, it could be a marker of the oxidative stress burden and the subsequent development of neurodegenerative disease^9^. To investigate the relationship between the serum GGT level and the risk of Parkinson’s disease (PD), we analyzed longitudinal big cohort data from the National Health Insurance Service (NHIS) database.

## Results

### Basic characteristics

We finally included a total of 6,098,405 individuals in the analysis. Baseline characteristics were compared among men and women in different quartiles of serum GGT activity (Table 1). There were factors that were significantly associated with serum GGT levels, e.g., age, low income, BMI, current smoking, mild-to-moderate alcohol consumption, exercise, diabetes mellitus (DM), hypertension, dyslipidemia, CKD, and metabolic syndrome (*p* < 0.0001). We considered all of these possible confounding variables when evaluating the independent relationship between GGT and PD.

**Table 1:** Baseline characteristics according to quartiles of serum GGT activity in men and women.

GGT (n)	Male					Female				
	Q1 (n = 701,522)	Q2 (n = 719,273)	Q3 (n = 734,048)	Q4 (n = 720,444)	*p*-value	Q1 (n = 848,532)	Q2 (n = 744,609)	Q3 (n = 847,154)	Q4 (n = 782,823)	*p*-value
GGT, IU/L	17.38	27.05	40.51	90.44	**<0.0001**	11.78	16.39	21.78	41.86	**<0.0001**
Age, yr	55.6 ± 11.4	54.6 ± 10.5	53.6 ± 10.0	52.5 ± 9.4	**<0.0001**	52.8 ± 10.7	54.5 ± 10.8	55.9 ± 10.7	56.6 ± 10.2	**<0.0001**
BMI, kg/m^2^	22.9 ± 2.6	23.9 ± 2.7	24.6 ± 2.8	25.0 ± 2.9	**<0.0001**	22.8 ± 2.7	23.4 ± 2.9	24.1 ± 3.1	24.9 ± 3.4	**<0.0001**
^a^Low income	22.4	22.2	22.3	23.3	**<0.0001**	32.1	31.6	31.2	31.3	**<0.0001**
Current smoker	29.7	34.3	38.7	45.2	**<0.0001**	1.7	2.2	2.8	4.3	**<0.0001**
^b^Mild-moderate drinking	43.7	54.1	63.5	75.1	**<0.0001**	15.9	17.5	18.6	21.5	**<0.0001**
^c^Exercise	55.9	57.3	57.3	56.7	**<0.0001**	44.2	43.2	42.2	40.6	**<0.0001**
DM	9.7	11.5	14.2	18.6	**<0.0001**	4.5	6.5	10.2	16.5	**<0.0001**
HTN	27.3	32.4	37.3	43.3	**<0.0001**	20.6	27.5	35.1	43.0	**<0.0001**
DL	12.2	18.1	23.5	28.9	**<0.0001**	14.0	21.0	28.5	37.1	**<0.0001**
CKD	6.4	6.5	6.5	5.8	**<0.0001**	7.0	8.3	9.7	10.7	**<0.0001**
MetS	17.5	26.9	36.4	47.1	**<0.0001**	18.5	27.8	39.2	52.1	**<0.0001**

Data are described as percentage of the individual groups, mean ± standard deviation (age, BMI), or geometric mean (GGT).

^a^low income defined as the lower quartile of income, ^b^mild to moderate drinking defined as alcohol consumption <30 g/day, ^c^exercise done for at least 20 minutes ≥1 time/week.

GGT, gamma-glutamyltransferase; BMI, body mass index; DM, diabetes mellitus; HTN, hypertension; DL, dyslipidemia; CKD, chronic kidney disease; MetS, metabolic syndrome; Q1, quartile 1; Q2, quartile 2; Q3, quartile 3; Q4, quartile 4.

### Baseline serum GGT activities and their impact on PD risk

**Figure 1:**
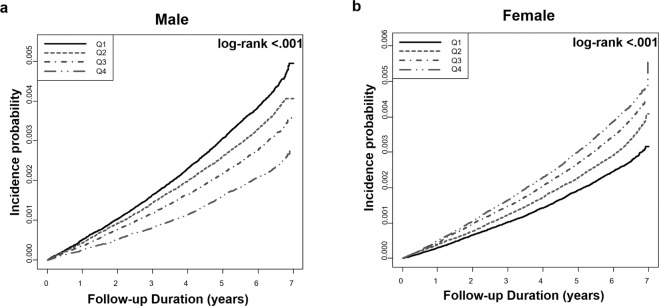
Probability of incident PD by GGT quartiles in men and women. Baseline serum GGT levels differentially predict PD development in men (**a**) and women (**b**). PD, Parkinson’s disease; GGT, gamma-glutamyltransferase, Q1, quartile 1; Q, quartile 2; Q3, quartile 3; Q4, quartile 4.

During the median follow-up of 6.4 years, new-onset PD developed in 20,895 participants (0.34%; 9,512 (0.33%) men and 11,383 (0.35%) women). The incidence of PD gradually decreased in men, while it increased in women as GGT activity increased (Table 2). The highest quartile of the GGT group had HRs for PD of 0.67 (95% CI = 0.63–0.71) in men and 1.33 (95% CI = 1.26–1.40) in women, using the lower three quartiles as a reference after adjustment for age. A regression model with adjustment for all possible confounders, including age, income, BMI, smoking, alcohol consumption, and exercise, still showed a significant impact of GGT on PD risk; the highest quartile of the GGT group had an adjusted HR for PD of 0.72 (95% CI = 0.67–0.76) in men and 1.30 (95% CI = 1.23–1.37) in women. Figure 1 presents a Kaplan-Meyer curve analysis for the probability of incident PD by each quartile of GGT in men and women. The probability decreased in men and increased in women from Q1 to Q4 of serum GGT activity (log-rank test *p* < 0.001). We created a table of numbers at risk by time separately (Supplementary Table S1). Additionally, we analyzed the sex-specific relationship between serum GGT activity and PD incidence in age groups by 10-year intervals. The relationship was consistently observed in all age groups except for men aged 80 years or above (Supplementary Fig. S1).

**Table 2:** Adjusted hazard ratios for incident PD according to serum GGT activity.

GGT (n)		Male				Female			
		Q1 (701,522)	Q2 (719,273)	Q3 (734,048)	Q4 (720,444)	Q1 (848,532)	Q2 (744,609)	Q3 (847,154)	Q4 (782,823)
Incident PD (n)		2,984	2,624	2,258	1,646	2,304	2,458	3,274	3,347
^a^Incidence rate		66.6	57.2	48.3	35.9	42.5	51.6	60.4	66.8
HR (95% CI)	^b^Model I	1	0.97 (0.92,1.02)	0.84 (0.80,0.89)	0.67 (0.63,0.71)	1	1.16 (1.10,1.23)	1.25 (1.18,1.31)	1.33 (1.26,1.40)
	^c^Model II	1	0.97 (0.92,1.02)	0.87 (0.82,0.92)	0.72 (0.67,0.76)	1	1.15 (1.08,1.21)	1.21 (1.15,1.28)	1.30 (1.23,1.37)

^a^PD incidence per 1,000 person-years, ^b^adjusted for age, ^c^adjusted for age, BMI, income, smoking, alcohol drinking, and exercise; income categorized as the lower quartile of income and the others; smoking and alcohol drinking as yes or no; doing exercise defined as at least 20 minutes ≥1 time/week.

PD, Parkinson disease; GGT, gamma-glutamyltransferase; HR, hazard ratio; CI, confidence interval; Q1, quartile 1; Q2, quartile 2; Q3, quartile 3; Q4, quartile 4.

### Interactions between serum GGT and obesity or metabolic syndrome and the risk of PD

In our population, both obesity and metabolic syndrome increased PD risk in both sexes (Tables 3 and 4), which was in line with previous reports^10^. Interestingly, the impact of both obesity and metabolic syndrome was higher in women than in men (adjusted HR = 1.22 (95% CI: 1.17–1.28) vs. 1.10 (95% CI: 1.05–1.15) and adjusted HR = 1.69 (95% CI: 1.61–1.77) vs. 1.34 (95% CI: 1.28–1.41) for women and men, respectively). We summarized the analysis results of the effect of the possible interaction between serum GGT and the presence of obesity or metabolic syndrome on the risk of PD in Tables 3 and 4. There was no interaction between GGT and obesity in men (*p* for interaction = 0.654), but we found a significant interaction between GGT and obesity in women (*p* for interaction = 0.005). The two factors increased PD risk in a subadditive manner (HR = 1.30 for obese women with Q4 GGT compared to nonobese women in Q1-Q3 GGT). When we conducted an interaction analysis of the effect of the association between serum GGT and the presence of metabolic syndrome on the risk of PD (Table 4), we found no interaction between the two factors in either sex (*p* for interaction = 0.654 and 0.457 in men and women, respectively).

**Table 3:** Impact of serum GGT level on PD risk by the presence of obesity.

^a^Obesity	GTP	Male				*p* for interaction	Female				*p* for interaction
		PD (n)	^b^IR	^c^MODEL I	^d^MODEL II		PD (n)	IR	MODEL I	MODEL II	
Non-obese	Q1-Q3	1,462,522	56.9	1	1	0.654	1,782,904	45.9	1	1	0.005
	Q4	369,356	38.5	0.72 (0.67,0.77)	0.75 (0.69,0.80)		427,301	60.7	1.19 (1.13,1.26)	1.20 (1.13,1.26)	
Obese	Q1-Q3	692,321	58.0	1.14 (1.09,1.20)	1.10 (1.05,1.15)		657,391	66.6	1.24 (1.18,1.30)	1.22 (1.17,1.28)	
	Q4	351,088	33.2	0.78 (0.72,0.84)	0.80 (0.74,0.86)		355,522	74.1	1.31 (1.24,1.39)	1.30 (1.23,1.38)	

^a^Obese subjects based on body mass index ≥25 kg/m^2^, ^b^PD incidence per 1,000 person-years, ^c^adjusted for age, ^d^adjusted for age, BMI, income, smoking, alcohol drinking, and exercise; income categorized as the lower quartile of income and the others; smoking and alcohol drinking as yes or no; doing exercise defined as at least 20 minutes ≥1 time/week.

GGT, gamma-glutamyltransferase; PD, Parkinson disease; IR, incidence rate; HR, hazard ratio; CI, confidence interval; Q1, quartile 1; Q2, quartile 2; Q3, quartile 3; Q4, quartile 4.

**Table 4:** Impact of serum GGT level on PD risk by the presence of metabolic syndrome.

Metabolic syndrome	GTP	Male				*p* for interaction	Female				*p* for interaction
		PD (n)	^a^IR	^b^MODEL I	^c^MODEL II		PD (n)	IR	MODEL I	MODEL II	
No	Q1-Q3	1,571,317	48.3	1	1	0.654	1,744,781	34.4	1	1	0.457
	Q4	381,167	30.1	0.67 (0.62,0.73)	0.73 (0.67,0.79)		374,765	39.2	1.06 (0.99,1.14)	1.10 (1.02,1.18)	
Yes	Q1-Q3	583,526	81.1	1.44 (1.37,1.50)	1.34 (1.28,1.41)		695,514	94.1	1.76 (1.69,1.84)	1.69 (1.61,1.77)	
	Q4	339,277	42.5	0.95 (0.89,1.02)	1.00 (0.93,1.08)		408,058	92.0	1.82 (1.73,1.91)	1.79 (1.70,1.89)	

aPD incidence per 1,000 person-years, ^b^adjusted for age, ^c^adjusted for age, BMI, income, smoking, alcohol drinking, and exercise; income categorized as the lower quartile of income and the others; smoking and alcohol drinking as yes or no; doing exercise defined as at least 20 minutes ≥1 time/week.

PD, Parkinson disease; GGT, gamma-glutamyltransferase; IR, incidence rate; HR, hazard ratio; CI, confidence interval; Q1, quartile 1; Q2, quartile 2; Q3, quartile 3; Q4, quartile 4.

## Discussion

To our knowledge, this is the first big data analysis demonstrating a significant association between serum GGT level and the future development of PD. In this study, the impact of serum GGT on the risk of PD was different between men and women. We observed that men with higher serum GGT activity had a lower risk of PD development, whereas women with higher serum GGT activity had a higher risk of developing PD, independent of age, income, BMI, smoking, alcohol consumption, and exercise. The relationship between GGT and PD remained significant regardless of the presence of obesity and metabolic syndrome.

Serum GGT values vary by sex and age group^11^. Direct release in the context of hepatocyte injury is not the sole cause of serum GGT elevation. GGT elevation also occurs due to the increased expression, decreased breakdown, or the release of enzymes from the cell membrane into circulation through unknown mechanisms^1^. Many factors have been known to relate to serum GGT activities, including obesity, DM, and components of metabolic syndrome^2,5,12,13^. Furthermore, GGT is related to the outcomes of a neurodegenerative disease^3^. The biological mechanism of serum GGT in these conditions is suggested to be associated with inflammation and oxidative stress^9,14^. Because abnormal protein aggregation, mitochondrial dysfunction resulting in oxidative stress, and neuroinflammation are the possible pathogenic mechanism of PD^15^, GGT may be linked to the risk of PD development through inflammatory and oxidative stress reactions.

However, the general hypothesis could not explain the contrary predictive effects of GGT on PD risk between men and women. Although sex-dependent factors such as health behaviors, including smoking, alcohol consumption, and exercise, may have a differential influence on disease risk, estrogen has been consistently thought to be a biological factor explaining the disparity between men and women^16–18^. Several studies have suggested that estrogen may play a protective role in the development of PD^19,20^, and plasma GGT activities were shown to be inversely associated with estrogen in an ovariectomized mouse model^21^ and in humans undergoing hormonal replacement therapy^22^. We speculated that this could partially explain the sex-specific relationship between GGT and PD.

Uric acid (UA) has been found to have a protective effect on PD development only in men, and this effect has also been shown to have sex differences^23–26^. Serum UA was not included in the Korean national health screening program; thus, we were not able to evaluate UA for the risk of PD development in this study. The levels of serum UA, an important natural antioxidant, may be parallel with serum GGT levels due to their relationship with oxidative stress, as reported in the general population and diabetic patients^27,28^.

Because the GGT level may be related to the presence of fatty liver, which is linked to obesity and metabolic syndrome, we conducted further analysis of the effect of the possible interaction between these factors on the risk of PD. The sex-specific impact of GGT remained significantly related to the presence of obesity in both men and women. Interestingly, there was no significant effect of the interaction between GGT and obesity on PD development in men, whereas in women, a significant effect of the interaction between GGT and obesity was observed. Obese women tended to have an even higher risk of PD development. This interaction suggests some degree of interrelationship between the GGT level and obesity in women. Unlike women, men with higher GGT levels might be less exposed to other risk factors for PD; thus, the net effect is more likely to be toward a reduced risk, whereas the higher GGT level is less likely to be related to obesity or metabolic syndrome itself in men. Furthermore, weight loss that could occur during the early course of PD^29,30^ might affect the risk estimation because this study was conducted with a relatively short follow-up period (median 6.4 years); therefore, the individuals who had already developed pathological changes in their body but were diagnosed with PD only after developing overt clinical signs later, might have been included in the initial population, even though we applied a 1-year lag period.

Several prospective cohort studies reported the relationship between metabolic syndrome and the future risk of PD^10,31^. One nationwide cohort analysis showed that metabolic syndrome increased the risk of PD. Stratified by metabolic syndrome status, the association between GGT and the risk of PD remained significant, and the effects of the interactions between GGT and metabolic syndrome on the risk of PD were unclear in both men and women.

The study cohort was restricted to the Korean population, and further validations are necessary in different ethnic cohorts. Because of the observational study design, the possible impact of serum GGT activity on PD development could not indicate causal relationships, and we relied on the operational diagnosis of PD without pathological confirmation. Despite these limitations, the present study was a longitudinal analysis of big data and revealed a novel association between GGT activity and the future development of PD, and GGT may be a candidate plasma biomarker that can predict or modify the risk of PD. The current analysis has methodological strength because it used nationwide cohort data, which could minimize bias from recall, selection, and hospital accessibility, and we adjusted for the effects of known risk factors for PD in our statistical model in the estimation of HR based on the basal GGT level.

In summary, this nationwide big data cohort analysis showed that the serum GGT level has a significant impact on the risk of PD, with a differential association in men and women. Further studies are warranted to investigate the validity of serum GGT as a marker predicting PD risk in other ethnic populations.

## Methods

### Data source

To avoid potential selection bias, we utilized the Korean NHIS big database because it has been mandatory for all citizens to register in the NHIS in Korea since 1989^8,32^. Korean people aged over 40 (or 30 in cases of cervical cancer screening) are recommended to receive a health screening every two years in government-authorized centers, and the examination rate is above 70% of the entire population^33^. This program data were also registered in the NHIS database, which includes demographic information (e.g., age, sex, and income level), questionnaires on health habits (e.g., smoking status, alcohol consumption, and exercise), body measurement (e.g., height, weight, and waist circumference), and cardio-cerebrovascular risk factors (e.g., DM, hypertension, dyslipidemia, ischemic heart disease, and stroke) based on history taking and evaluations (e.g., blood pressure (BP), blood samples including fasting blood glucose (FBG), creatinine, lipid panels, and liver enzymes). In this study, we determined the presence of metabolic syndrome and chronic kidney disease (CKD) following the criteria shown in the supplementary material (Supplementary Note).

### Study population

In 2009, a total of 10,505,818 individuals received a health check-up. Among them, individuals under 40 years old (n = 3,322,546, 31.6%), with heavy alcoholism (defined as 30 g/day, n = 491,241, 4.7%), with missing data for any variables (n = 291,174, 2.8%), with hepatobiliary and pancreatic diseases including cancer and viral hepatitis (n = 288,096, 2.7%), and with a history of PD (n = 11,691 before the study commenced and n = 2,665 in the first 1 year of the follow-up period, 0.1%) were excluded in this study. We followed the cohort and recorded newly diagnosed PD until December 31, 2016. The Institutional Review Board (IRB) of Seoul National University-Seoul Metropolitan Government Boramae Medical Center approved this study (IRB No. 07-2018-14), and we performed the study in accordance with the relevant guidelines and regulations. The study analyzed the national big data under strict regulation following the procedures of the country, and the IRB waived the requirement for written informed consent due to the use of anonymous and deidentified information.

### Identification of incident PD

The Korean government launched a legal support system for PD as it is categorized as a rare incurable disease in Korea, so all PD patients are required to register in the governmental insurance system with a diagnostic code G20 by International Classification of Diseases–10^th^ Revision–Clinical Modification (ICD-10-CM). We defined incident PD in this study when it was newly registered in the governmental system with diagnostic code G20 during the follow-up period after the exclusion of newly diagnosed PD cases during the first 1 year, which was considered the lag period. The Korean medical insurance system uses ICD-10-CM codes, which designate PD and the other parkinsonian syndromes as separate disease codes (e.g., vascular parkinsonism is classified as G214 and was not included in the study population).

### Statistical analysis

The study population was divided into 4 groups according to the quartiles of serum GGT levels, and the baseline characteristics of the groups were analyzed by the Kruskal-Wallis test for continuous variables (GGT, age, body mass index (BMI)) and by the chi-square test for categorical variables. The Cox regression analysis was used to calculate hazard ratios (HRs) with 95% confidence intervals (CIs) for PD development for each quartile of serum GGT. Covariates included age and BMI as continuous variables and sex, income, smoking, alcohol consumption, and exercise as categorical variables; income was categorized as the lower quartile of income and others; smoking and alcohol consumption as yes or no; and exercise was defined as performing at least 20 minutes of exercise ≥1 time/week. To test the interaction between GGT and other risk factors, the HR for PD and the *p* for interaction were estimated in subjects grouped by obesity or metabolic syndrome status separately for men and women. In this analysis, obesity was defined as BMI ≥ 25 kg/m^2^, which is widely accepted in Korea according to the risk of mortality and the WHO recommendations^34–36^. Statistical analyses were performed using SAS version 9.4 (SAS Institute, Cary, NC, USA) and R version 3.2.3 (The R Foundation for Statistical Computing, Vienna, Austria; http://www.Rproject.org) with the significance level set at 0.05 (two tailed).

## Supplementary information

Supplementary information.

## Data Availability

The Korean NHIS database are confidential and approved to use for researchers who meet the criteria for access through the Korea National Health Insurance Sharing Service (NHISS) Institutional Data Access Committee (https://nhiss.nhis.or.kr/bd/ay/bdaya001iv.do). If data are requested for additional analysis, the corresponding author would consider it deliberately to offer after passing the review process of the Korea NHISS Institutional Data Access Committee, and after payment data access fee charged to the requester.
