# Supplementary material for: Serum gamma-glutamyltransferase activity and Parkinson’s disease risk in men and women
Source: Sci Rep. 2020 Jan 27;10:1258. doi: 10.1038/s41598-020-58306-x (PMC6985223; doi:10.1038/s41598-020-58306-x)
Supplement: Supplementary file 1 — Supplementary information. [file 41598_2020_58306_MOESM1_ESM.docx]

Title:

**Serum gamma-glutamyltransferase activity and Parkinson’s disease risk in men and women**

Dallah Yoo, MD^1, 2^, Ryul Kim, MD^1, 2^, Yu Jin Jung, MD, PhD^3^, Kyungdo Han, MD, PhD^4^, Cheol Min Shin, MD, PhD^5^, Jee-Young Lee, MD, PhD^1, 2*^

^1^Department of Neurology, Seoul National University-Seoul Metropolitan Government Boramae Medical Center, Seoul, Republic of Korea

^2^Department of Neurology, Seoul National University College of Medicine, Seoul, Republic of Korea

^3^Department of Neurology, Daejeon St. Mary’s Hospital, College of Medicine, The Catholic University of Korea, Daejeon, Republic of Korea

^4^Department of Medical Statistics, College of Medicine, The Catholic University of Korea, Seoul, Republic of Korea

^5^Department of Internal Medicine, Seoul National University Bundang Hospital, Seongnam, Gyeonggi, Republic of Korea

***Correspondence:** Jee-Young Lee, MD, PhD.

Department of Neurology, Seoul National University College of Medicine & Seoul Metropolitan Government-Seoul National University Boramae Medical Center

20 Boramae-ro 5-gil, Dongjak-gu, Seoul, 07061, Republic of Korea

Tel: +82-2-870-2476 Fax: +82-2-870-2853 E-mail: wieber04@snu.ac.kr

**Supplementary Note. Definition of metabolic syndrome and chronic kidney disease in this analysis**

We defined metabolic syndrome if at least 3 of the five criteria were met; (i) WC≥90 cm for men or ≥85 cm for women; ^1^ serum triglycerides≥1.70 mmol/l or treatment with lipid-lowering medication at least 1 claim of prescription per year under International Classification of Diseases–10th Revision–Clinical Modification (ICD-10-CM) code E78; (iii) serum high-density lipoprotein cholesterol (HDL-C)<1.04 mmol/l for men or <1.30 mmol/l for women or treatment with lipid-lowering medication; (iv) systolic BP≥130 mm Hg, diastolic BP≥85 mm Hg, or treatment with antihypertensive medication; and (v) FBG≥5.55 mmol/l or use of hypoglycemic agents. Estimated glomerular filtration rate (eGFR) was calculated using the equation from the Modification of Diet in Renal Disease (MDRD) study: eGFR = 175 × serum creatinine^−1.154^ × age^−0.203^, further multiplied by 0.742 for women^2^. We defined eGFR<60 mL/min/1.73m^2^ as chronic kidney disease (CKD)^3^.

1 Yamada, J. *et al.* Elevated serum levels of alanine aminotransferase and gamma glutamyltransferase are markers of inflammation and oxidative stress independent of the metabolic syndrome. *Atherosclerosis* **189**, 198-205, doi:10.1016/j.atherosclerosis.2005.11.036 (2006).

2 Levey, A. S., Coresh, J., Greene, T. & et al. USing standardized serum creatinine values in the modification of diet in renal disease study equation for estimating glomerular filtration rate. *Annals of Internal Medicine* **145**, 247-254, doi:10.7326/0003-4819-145-4-200608150-00004 (2006).

3 Levey, A. S., Coresh, J., Balk, E. & et al. National kidney foundation practice guidelines for chronic kidney disease: Evaluation, classification, and stratification. *Annals of Internal Medicine* **139**, 137-147, doi:10.7326/0003-4819-139-2-200307150-00013 (2003).

**Supplementary Table S1. The number of patients at risk in Kaplan-Meier curve of PD development according to serum GGT**

Numbers at risk are shown by time in the Kaplan-Meier curve of future PD development according to quartiles of baseline serum GGT levels. PD, Parkinson disease; GGT, gamma-glutamyltransferase; Q1, quartile 1; Q2, quartile 2; Q3, quartile 3; Q4, quartile 4.

|  | GTP | Follow-up duration (years) | | | | | | | |
| --- | --- | --- | --- | --- | --- | --- | --- | --- | --- |
| Total |  | **0** | **1** | **2** | **3** | **4** | **5** | **6** | **7** |
|  | **Q1** | 1,550,054 | 1,549,434 | 1,548,764 | 1,548,026 | 1,547,221 | 1,546,266 | 1,545,263 | 0 |
|  | **Q2** | 1,463,882 | 1,463,276 | 1,462,641 | 1,461,926 | 1,461,164 | 1,460,303 | 1,459,337 | 0 |
|  | **Q3** | 1,581,202 | 1,580,555 | 1,579,814 | 1,579,080 | 1,578,249 | 1,577,313 | 1,576,237 | 1 |
|  | **Q4** | 1,503,267 | 1,502,688 | 1,502,063 | 1,501,390 | 1,500,645 | 1,499,740 | 1,498,733 | 0 |
| Male |  | **0** | **1** | **2** | **3** | **4** | **5** | **6** | **7** |
|  | **Q1** | 701,522 | 701,159 | 700,795 | 700,368 | 699,901 | 699,370 | 698,821 | 0 |
|  | **Q2** | 719,273 | 718,959 | 718,607 | 718,232 | 717,836 | 717,397 | 716,899 | 0 |
|  | **Q3** | 734,048 | 733,781 | 733,488 | 733,174 | 732,835 | 732,442 | 732,013 | 1 |
|  | **Q4** | 720,444 | 720,257 | 720,063 | 719,856 | 719,616 | 719,278 | 718,941 | 0 |
| Female |  | **0** | **1** | **2** | **3** | **4** | **5** | **6** | **7** |
|  | **Q1** | 848,532 | 848,275 | 847,969 | 847,658 | 847,320 | 846,896 | 846,442 | 0 |
|  | **Q2** | 744,609 | 744,317 | 744,034 | 743,694 | 743,328 | 742,906 | 742,438 | 0 |
|  | **Q3** | 847,154 | 846,774 | 846,326 | 845,906 | 845,414 | 844,871 | 844,224 | 0 |
|  | **Q4** | 782,823 | 782,431 | 782,000 | 781,534 | 781,029 | 780,462 | 779,792 | 0 |

**Supplementary Figure S1. Baseline serum GGT activity and incidence of PD by sex and age**

The incidence rates of PD in the two subgroups of GGT activities are plotted by age in men and women. The upper quartile (Q4) of GGT shows lower incidence of PD than the others (Q1-Q3) in all age groups except for the aged 80 or above in men. In women, the upper quartile (Q4) of GGT shows higher incidence of PD than the others (Q1-Q3) in all age groups.

Parkinson disease; GGT, gamma-glutamyltransferase; Q1, quartile 1; Q2, quartile 2; Q3, quartile 3; Q4, quartile 4.
